# Identification and validation of mRNA profiles linked to ATP- induced cell death represent a novel prognostic model for breast cancer

**DOI:** 10.3389/fimmu.2024.1483498

**Published:** 2024-11-01

**Authors:** Zhongwen Zhang, Haolong Zhang, Zheng Zhang, Doblin Sandai, Ping Lu, Haoling Zhang, Jianjun Wu

**Affiliations:** ^1^ School of Public Health, Gansu University of Chinese Medicine, Lanzhou, Gansu, China; ^2^ Department of Biomedical Sciences, Advanced Medical and Dental Institute, Universiti Sains Malaysia, Kepala Batas, Penang, Malaysia; ^3^ The first affiliated hospital of Xinxiang medical university, Xinxiang, Hean, China

**Keywords:** ATP-induced cell death, prognostic model, breast cancer, drug sensitivity, immunological assessment, personalized treatment

## Abstract

**Background:**

Cell death mechanisms are integral to the pathogenesis of breast cancer (BC), with ATP-induced cell death (AICD) attracting increasing attention due to its distinctive specificity and potential therapeutic applications.

**Methods:**

This study employed genomic methodologies to investigate the correlation between drug sensitivity and types of AICD in BC. Initially, data from TCGA were utilized to construct a prognostic model and classification system for AICD. Subsequently, a series of bioinformatics analyses assessed the prognostic and clinical significance of this model within the context of BC.

**Results:**

Analysis revealed a cohort of 18 genes associated with AICD, exhibiting prognostic relevance. Survival analyses indicated that overall survival rates were significantly lower in high-risk populations compared to their low-risk counterparts. Furthermore, prognostic indicators linked to AICD demonstrated high accuracy in predicting survival outcomes in BC. Immunological assessments indicated heightened expression of anti-tumor infiltrating immune cells and immune checkpoint molecules in low-risk populations, correlating with various anti-tumor immune functions. Ultimately, a comprehensive prognostic model related to AICD was developed through univariate analysis, least absolute shrinkage and selection operator (LASSO), and multivariate Cox regression analysis. As Adenosine triphosphate (ATP) concentration increased, the viability of BC cells exhibited a general decline at each time point. Notably, ATP diminished the mitochondrial membrane potential in BC cells while enhancing it in normal breast epithelial cells. Additionally, ATP inhibited the migration of BC cells and promoted their apoptosis. ATP also stimulated reactive oxygen species (ROS) production in MCF-10A cells, with implications for the immune response in BC cells. Compared to the control group, expression levels of *CLIC6*, *SLC1A1*, and *CEMIP* were significantly reduced in the ATP intervention group, whereas *ANO6* expression was elevated. *ANO6*, *CEMIP*, and *CLIC6* share genetic variants with BC, while *SLC1A1* does not exhibit genetic causal variation with the disease.

**Conclusion:**

A valuable prognostic model associated with AICD has been established, capable of accurately predicting BC prognosis. The induction of cell death by ATP appears to play a protective role in BC progression. These findings carry significant implications for the implementation of personalized and tailored treatment strategies for BC patients.

## 1 Introduction

Breast cancer (BC) remains the leading cause of cancer-related fatalities among women, highlighting its complex disease variations, metastatic tendencies, and resistance to treatment (1). According to the International Agency for Research on Cancer (IARC), BC accounts for 11.7% of all cancer cases globally, solidifying its status as the most prevalent cancer type worldwide. Research into BC continues to be a focal point both at home and abroad. Despite advancements in early detection and treatment modalities, formidable challenges persist due to unfavorable prognoses faced by patients. Consequently, the exploration of novel prognostic models and the identification of emerging biomarkers for BC necessitate substantial attention and investigation.

Adenosine triphosphate functions as a multifaceted signaling molecule within both intracellular and extracellular environments, exerting a pivotal influence on cellular energy metabolism and intricately intertwining with the regulation of cellular functions and the onset of diseases. While previous investigations have primarily focused on the release and effects of ATP on neuronal activity and neurotransmission, recent research has revealed its capacity to induce cell demise, termed ATP-induced cell death (AICD), particularly under conditions of elevated extracellular ATP levels (2). AICD, recognized as a novel form of cellular demise in 1991, is characterized by substantial accumulation of ATP and activation of ATP membrane receptors during the cell death process, hence the designation AICD (3). Hao-Ling explores the dynamic interplay between the cellular system and ATP homeostasis, elucidating the intricate mechanisms governing AICD, detailing its involvement in cancer signaling pathways, and meticulously examining the pivotal genes implicated in this process (4). Cell death mechanisms play a crucial role in the pathogenesis of BC, with ATP-triggered cell death attracting increasing attention due to its distinctive specificity and potential therapeutic ramifications. These inquiries suggest that AICD may hold promise for the treatment of BC; however, the correlation between AICD-related genes and BC prognosis remains unclear. Recent AICD mRNA prognostic models incorporate four specific mRNAs: P2X purine receptor 4, pannexin 1, caspase 7, and cyclin 2, while the amalgamated miRNA prognostic model integrates four pivotal miRNAs: hsa-miR-615-3p, hsa-miR-519b-3p, hsa-miR-342-3p, and hsa-miR-324-3p, successfully constructing prognostic frameworks for BC. This elucidates the molecular underpinnings of AICDs in BC, enhancing the arsenal of potential therapeutic modalities and providing an unprecedented avenue for innovative interventions (5). In essence, this paper unveils a previously enigmatic link between AICD and BC that could herald revolutionary strides in personalized oncology. However, the expression profiles of miRNA and mRNA are typically derived from disparate experimental platforms or sample sources, and integrating such data may pose challenges due to significant diversity, potentially compromising the stability and reliability of the model. Therefore, prospective experimental validation is imperative to enhance the credibility of the study findings and mitigate potential limitations in data interpretation. This study distinguishes itself from prior research through the subcompositional analysis and genotyping of BC, thereby enhancing the model’s accuracy and validating its findings.

AICD, Ferroptosis, and Cuproptosis represent the three distinct mechanisms of cellular demise, each intricately linked to energy metabolism and the biochemical roles of iron and copper, respectively. Ferroptosis constitutes an iron-dependent form of cell death, primarily resulting from the accumulation of lipid peroxides. The presence of iron ions facilitates the Fenton reaction, generating free radicals that instigate lipid oxidation. Conversely, Cuproptosis, a recently identified mechanism, is closely associated with the accumulation of copper ions within cells. By interacting with mitochondria, copper ions induce oxidative stress, culminating in cellular death. Notably, during the occurrence of iron death and copper death, a significant release of ATP occurs outside the cell, thereby instigating AICD. Thus, AICD serves as the fundamental mechanism linking iron death and copper death, underscoring the necessity for a comprehensive understanding of these cell death pathways in the context of BC.

In the realm of BC research, the selection of cell lines holds significant scientific importance, as distinct cell lines exhibit notable differences in their biological characteristics, gene expression profiles, and responses to therapeutic interventions. This study employs three representative cell lines: MCF-7, MDA-MB-231, and MCF-10A. The MCF-7 cell line is classified as hormone receptor-positive (HR+) and HER2-negative (HER2-), and it is extensively utilized to investigate the biological properties of hormone-dependent tumors and their associated treatment strategies. In contrast, the MDA-MB-231 cell line epitomizes triple-negative BC (TNBC), whose pronounced invasiveness and metastatic potential render it a crucial model for exploring tumor progression and mechanisms of resistance. Furthermore, the MCF-10A cell line, which comprises non-tumorigenic breast epithelial cells, serves as a control cell line that facilitates a deeper understanding of the biological distinctions between normal breast epithelial cells and malignant counterparts. Through a comprehensive examination of these three cell lines, we aim to elucidate the complexities of BC and provide novel insights into potential therapeutic targets.

Therefore, gene expression profiles and corresponding clinical data of BC patients were obtained from the Cancer Genome Atlas (TCGA) database. Gene expression patterns associated with AICD were described, followed by subcompositional analysis and genotyping of BC patients. Risk scores were calculated to identify BC prognosis, and the prognostic efficacy was validated to enhance the model’s accuracy. Additionally, underlying mechanisms in high-risk (HR) and low-risk (LR) populations were explored through gene set enrichment analysis (GSEA) and immune microenvironment assessment. Finally, these findings were confirmed and elaborated upon at the single-cell level.

## 2 Materials and methods

### 2.1 Transcriptome data download

Transcriptome data of BC, along with relevant clinical information and mutation data, were obtained from the TCGA database (https://portal.gdc.cancer.gov/), facilitating the extraction of pertinent information. AICD genes were previously reported by Zhang H L (Haoling Zhang)? in the literature[5.6]. The literature search aimed to identify core genes associated with AICD using the following keywords: AICD, cell death, apoptosis, autophagy, necrosis, death, and extracellular ATP. The search was conducted between March 10, 2023 and June 20, 2023. Databases including PubMed, Embase, Web of Science, MESH, and Scopus were employed to identify genes related to AICD. The scope of the search was expanded to encompass genes associated with AICD across all diseases, given the limited availability of literature reporting AICD specifically in BC. Publications that did not mention AICD were excluded from the analysis.

### 2.2 Differential analysis and mutation analysis of AICD in BC

The selected gene data underwent standardization using R language. The limma package facilitated gene screening based on differential expression, employing filter conditions of | logFC | > 1 and adj. P.val < 0.05 to identify significantly different genes. Tumor mutation burden (TMB) serves as a biomarker for assessing the frequency of gene mutations. Tumor cells exhibiting a high frequency of gene mutations present an increased number of tumor antigens on their surface, rendering them more susceptible to immune system attacks, ultimately leading to cell death. Calculations were primarily conducted through TMB scripts. Copy number data for AICD were extracted from the UCSC website (https://xena.ucsc.edu/), and copy number variations were analyzed using R packages, with concurrent genotyping analysis performed.

### 2.3 PCA and survival prognosis analysis

Based on the expression pattern of AICD multiomics, principal component analysis (PCA) was employed to effectively reduce dimensionality, facilitate model recognition, and visualize groupings within the complete gene expression profile and the high-dimensional data of the risk model. The Kaplan-Meier curve was utilized to evaluate overall survival (OS) differences between two distinct populations. The R package served as the primary tool for conducting this survival and prognostic analysis.

### 2.4 Enrichment analysis

The ‘clusterProfiler’ and ‘enrichment plot’ packages in R facilitated Gene Ontology (GO) analysis, KEGG analysis, and Gene Set Enrichment Analysis (GSEA) of differentially expressed genes (DEGs), with a significance threshold set at P < 0.05. GSEA was conducted using imminent gene sets from the Molecular Signatures Database (MsigDB), where P < 0.05 and false discovery rate (FDR) Q values < 0.05 were considered indicative of significant enrichment.

### 2.5 Establishment and proving of a predicted lipography

The predictive power of photographic assessments and additional variables (age, sex, risk score, TNM stage, T, N, M, and smoking history) for overall survival (OS) at 1, 3, and 5 years was established. Consistency between actual outcomes and model predictions was demonstrated through calibration curves based on Hosmer-Lemeshow testing.

### 2.6 Immunoassay

The R package was utilized to compare immunization and ESTIMATE scores across two distinct populations. Subsequently, single sample Gene Set Enrichment Analysis (ssGSEA) was performed using the “GSVA” and “GSEABase” R packages to evaluate various immune-related features, including infiltration scores of 16 immune cell types and activity levels of 13 immune-related pathways. Additionally, the R software package was employed to contrast the levels of immune infiltration between the two populations.

### 2.7 Exploration of compounds with clinical potentials

To identify novel compounds for clinical application in BC therapy, semi-inhibitory concentrations of candidate compounds extracted from the GDSC database within the TCGA BC dataset were calculated. R packages were employed to predict the semi-inhibitory concentrations of compounds derived from the GDSC database in the BC population.

### 2.8 Statistical analysis

All analyses were conducted using Perl software (version 5.32.0.1-64-bit; https://strawberryperl.com/) and R software (version 4.0.3; https://www.r-project.org). Within the various analyses, a p-value of < 0.05 was considered “statistically significant,” a p-value of < 0.01 was classified as “more statistically significant,” and a p-value of < 0.001 was designated as “most statistically significant”.

### 2.9 Cell viability was measured by CCK-8

Cell suspensions of MCF7, MDA-MB231, and MCF-10A cell lines were prepared and inoculated into each well of a 96-well plate, achieving a cell concentration of 1×10^4^-10^5^ per well. Sterile PBS buffer was added around each cell suspension well. The 96-well plate containing the cell suspension was then placed in a cell incubator set at 37°C with 5% CO2 for standard incubation. Upon cell adhesion, agents with ATP concentrations ranging from 0.1 to 5 mmol/L were introduced for intervention, with exposure durations of 15, 30, 45, 60, and 120 minutes to assess their impact on cell viability. Following the removal of the surface medium, 10 µL of CCK-8 solution (Shenzhen Sunview Technology Co., Ltd, Lot: DCM2188) was added directly to each well and incubated in the incubator for 2 hours. Subsequently, absorbance at 450 nm was measured using an enzyme-linked instrument. The concentration and duration of the intervention were finalized through screening, with the optical density (OD) value serving as the criterion for concentration differentiation in this study.

### 2.10 LPO content detection

The experiment was divided into a control group (without ATP) and an ATP-treated group. The previously determined culture medium treatment time was used to establish the appropriate ATP concentration for screening. Following the washing, digestion, and collection of cells, LPO detection (Solarbio science & technology (Bei Jing) Co., Ltd, Lot: NO.2309002) was carried out using a specific kit. Blank tubes, standard tubes, and test tubes were prepared accordingly, and samples were added as per the manufacturer’s instructions. The samples were mixed and incubated at 45°C for 60 minutes, after which the supernatant was obtained via centrifugation. The OD value was measured at a wavelength of 586 nm, and the LPO content was calculated using the following formula: LPO content (μmol· GPROt^-1^) = (A_test - A_blank)/(A_standard - A_blank) ×standard substance concentration (10 μmol·L^-1^) ÷ sample protein concentration (gprot· mL^-1^).

### 2.11 Determination of mitochondrial membrane potential

MCF7, MDA-MB231, and MCF-10A cells were cultured in confocal dishes at a cell density of 1×10^4^ cells per dish. The cells were treated according to the protocol outlined in section 2.10, followed by a single wash with PBS. Subsequently, 1 mL of cell culture medium was added, followed by 1 mL of JC-1 staining solution (Solarbio science & technology (Bei Jing) Co., Ltd, Lot: NO.2311005). After a 20-minute incubation with the working solution, the cells were rinsed using the specific buffer provided. Finally, 2 mL of cell culture medium was added to the cells, which were then observed under a laser confocal microscope.

### 2.12 Assessment of cell migration capacity using a scratch assay

Cells were seeded in 6-well plates and grouped as described in section 2.10. After the intervention, once the cells reached confluence and formed a monolayer, a scratch was made perpendicular to the horizontal axis using a sterile 20μL pipette tip. The cells were then washed three times with PBS, and serum-free culture medium was added. Images were captured using an optical microscope at 0 hours, and the cells were subsequently incubated. Additional images were taken at 24 and 48 hours using the same optical microscope. The results were analyzed using ImageJ software.

### 2.13 Apoptosis was evaluated through TUNEL staining analysis

Following the grouping and intervention of MCF7, MDA-MB231, and MCF-10A cells as outlined in section 2.10, 500µL of TUNEL staining solution was added to each well according to the TUNEL kit instructions (Biosharp, BL114A). The cells were incubated at 37°C for 1 hour in the dark. After thorough washing with PBS, 1µg/mL of DAPI solution was added and incubated at room temperature for 10 minutes. The residual liquid was carefully removed using tissue paper, and the cells were observed and imaged under a fluorescence microscope. Quantitative analysis was then conducted using ImageJ software.

### 2.14 Invasion

The matrix glue was naturally melted overnight at 4°C, and the microinjection gun and its tip (blue and yellow tip) used in the experiment were pre-frozen at -20°C. The matrix glue was prepared by mixing 2.5μl of Matrigel (Corning Incorporated,REF 354248)per well with 97.5μl of medium (without bovine serum and pre-stored at 4°C), gently mixed at the end of the gun and added to the central upper chamber of each 24-well Transwell plate with 100μl of mixture per well. Incubate at 37°C for 30 minutes to 1 hour. After the matrix glue was dried, normal digestion group and ATP intervention group cells, washed (3 times), and 100μl cells were suspended in the upper chamber, while 600μl medium containing 10% FBS was added to the lower chamber, and the culture was continued for 48 hours. After incubation, the liquid in the upper and lower chambers was discarded, washed 3 times with PBS, and then fixed with 4% paraformaldehyde(Lanzhou Kebao Biotechnology Co., Ltd.,35942) for 30 minutes. After fixing, it was washed with PBS for 3 times again, crystal violet (Lanzhou Kebao Biotechnology Co., Ltd.,3422) was added and it was dyed for 15 minutes. After staining, the chamber was cleaned with PBS and photographs were taken under a microscope.

### 2.15 Detection of apoptosis by flow cytometry

MCF7, MDA-MB231, and MCF-10A cells were cultured under optimal conditions and subsequently inoculated into 6-well plates at a density of approximately 1 × 10^5 cells per well, organized into groups of 2.10 cells. Following the intervention, the supernatant was removed, and the cells were digested using 0.5 mL of trypsin without EDTA. To terminate the digestion, 1 mL of complete medium (supplemented with 10% fetal bovine serum) was added. After a 5-minute incubation, the cells were precipitated, and the supernatant was discarded. The cells were then re-suspended in 1 mL of cell staining buffer, to which 5 µL of Hoechst staining solution and 5 µL of PI staining solution were added. The mixture was thoroughly mixed and incubated at 4°C for 20-30 minutes before analysis by flow cytometry (Biosharp, BL114A).

### 2.16 Detection of cell ROS by flow cytometry

Following the grouping of MCF7, MDA-MB231, and MCF-10A cells according to the 2.10 protocol and subsequent interventions, reactive oxygen species (ROS) levels in each group were assessed using a ROS detection kit (Beyotime, Cat NO. S0033S). The final concentration of DCFH-DA was set at 10 μmol/L by diluting DCFH-DA in serum-free medium at a ratio of 1:1000. The cell culture medium was discarded, and no less than 1 mL of the diluted DCFH-DA was added to each well. The cells were then incubated at 37°C for 20 minutes. After incubation, the cells were washed three times with serum-free cell culture medium, and Rosup was added to the positive control well to serve as the positive control.

### 2.17 RT-qPCR

RNA was extracted from each group using the Trizol method. Following the instructions provided by the reverse transcription kit, the total RNA was reverse transcribed to synthesize the first strand of complementary DNA (cDNA). The mRNA expression levels in the cells of each group were assessed using cDNA as the template. Employing GAPDH as the internal control, the relative mRNA expression levels in each group were quantified using the 2-ΔΔCt method. All mRNA primers utilized in this study were procured from Lanzhou Kebao Biotechnology Co., Ltd., 1174624162 with the primer sequences detailed in **Table 1**. Real-time fluorescent quantitative polymerase chain reaction (RT-qPCR) was employed to detect gene mRNA expression.

**Table 1:** Primer sequence.

Gene (M)	Prime	Product length	Login ID
ANO6	F: GGGATCACTGGAAAGTCAGCA	219	NM_001025356.3
	R: TGCAGGCCATGACAGATAAGG		
CEMIP	F: GGCCAATCCCAACAACAACC	179	NM_018689.3
	R: CCCGGTAGTTGGAATGTGCT		
CLIC6	F: CACGACATCACCCTCTTCGT	130	NM_053277.3
	R: AGAGACGCTGAGAAAACGGG		
SLC1A1	F: GAGGAAAGGATGCGAGTGGA	88	NM_004170.6
	R: TGTGGTAATGCCTAGCACCA		
GAPDH	F:GCAAAGTGGATGTTGTCGCC	132	NM_001082253.1
	R:TGATGACCAGCTTCCCGTTC		

### 2.18 Colocalization analysis of model gene and genetic variation of BC -eQTL-GWAS

Bayesian total positioning analysis was conducted to evaluate the probability that two phenotypes share the same causal variation, utilizing the default parameters of the “coloc” package (https://github.com/chr1swallace/coloc). The ‘ieugwasr_to_coloc’ function was employed to extract colocalization data, while the ‘coloc.abf’ function facilitated genetic colocalization analysis of the two potentially related phenotypes, examining whether they possessed common genetic causal variation within the genetic distance region associated with the corresponding eQTL gene. We established the truncation threshold for colocalization evidence as PP.H4.abf > 80%, and subsequently performed visualization using the ‘stack_assoc_plot’ function from the ‘gassocplot2’ package.

## 3 Results

### 3.1 Differential analysis and mutation analysis of AICD in BC

**Figure 1:**
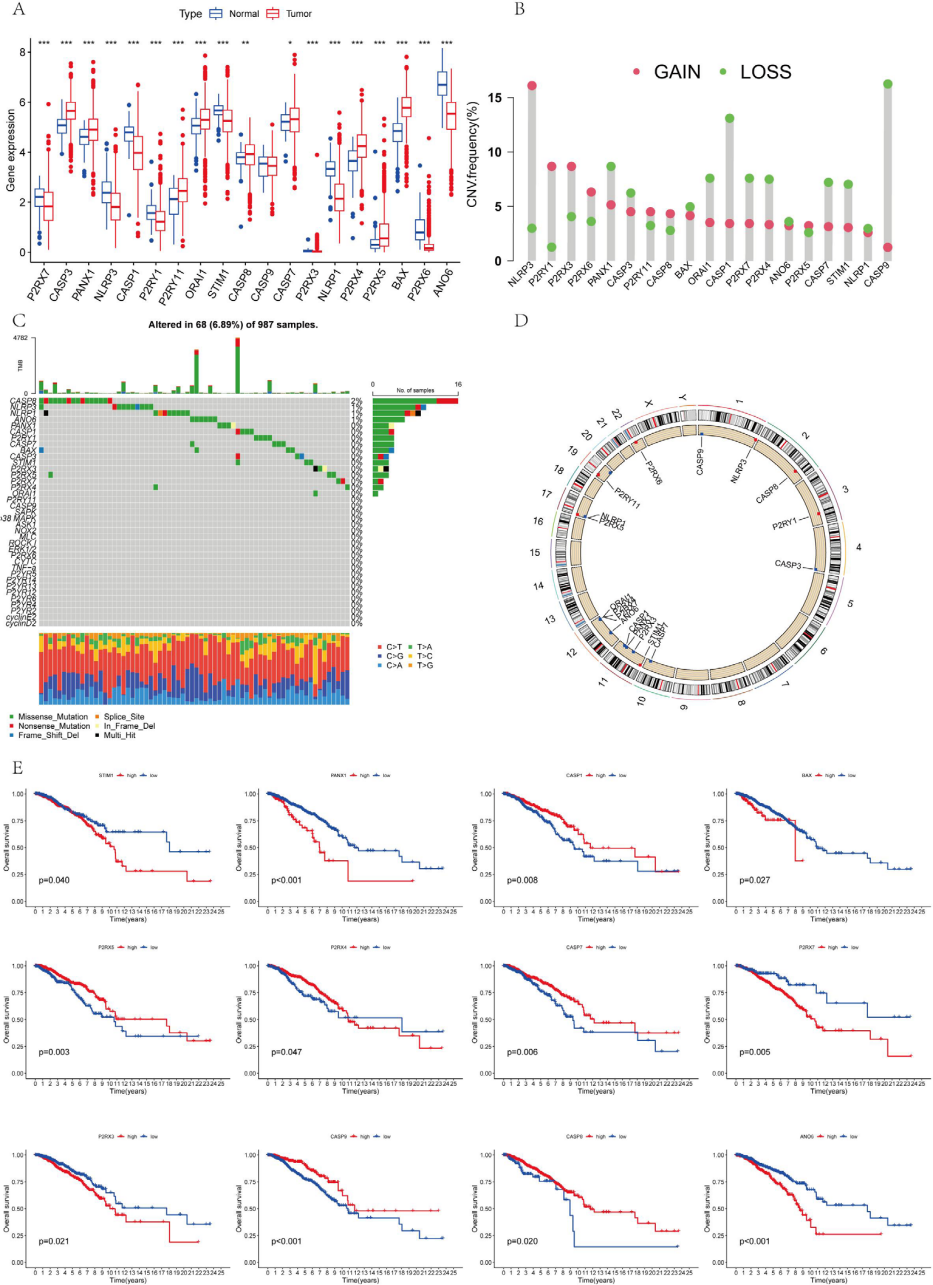
**(A)** The differential expression of AICD genes that is observed between normal and tumor cohorts in TCGA; **(B)** The distribution of CNV gain, loss, and non-CNV within AICD genes; **(C)** The frequency of copy number variation among AICD genes; **(D)** AICD genes located at CNV mutation sites across 23 chromosomes; **(E)** The correlation between the survival outcomes and AICD genes. *P < 0.05, **P < 0.01, ***P < 0.001.

**Figure 1A** illustrates the expression levels and mutation frequencies of genes associated with AICD within tumor tissues. Notably, the genes P2RX7, NLRP3, CASP1, P2RY1, STIM1, CASP9, P2RX3, NLRP1, P2RX6 and ANO6 genes were significantly down-regulated in these tissues (P <0.05). On the contrary, the expressions of CASP3, PANX1, P2RY11, ORAI1, CASP8, CASP7, P2RX5, P2RX4 and BAX were up-regulated. **Figure 1B** presents the frequency of copy number variations (CNV) in genes related to AICD in tumor tissues. **Figures 1B, D** reveal that the genes NLRP3, P2RY1, P2RX3, P2RX6, P2RY11, CASP8, and P2RX5 demonstrate a higher frequency of copy number amplification compared to copy number loss. In contrast, PANX1, CASP3, BAX, ORAI1, CASP1, P2RX7, P2RX4, ANO6, CASP7, STIM1, NLRP1, and CASP9 exhibit a greater frequency of copy number loss relative to amplification. **Figure 1C** details the mutation frequency of AICD-related genes in tumor tissue, where 68 out of 987 samples exhibited genetic mutations, resulting in a mutation frequency of 6.89%. The genes most frequently mutated were CASP8, NLRP3, NLRP1, and ANO6. In addition, a relationship was identified between AICD expression levels and prognostic variables in BC, suggesting the potential involvement of AICD in this disease. These biomarkers may serve as therapeutic targets or predictive indicators for BC.

### 3.2 AICD and survival analysis and prognosis analysis

**Figure 2:**
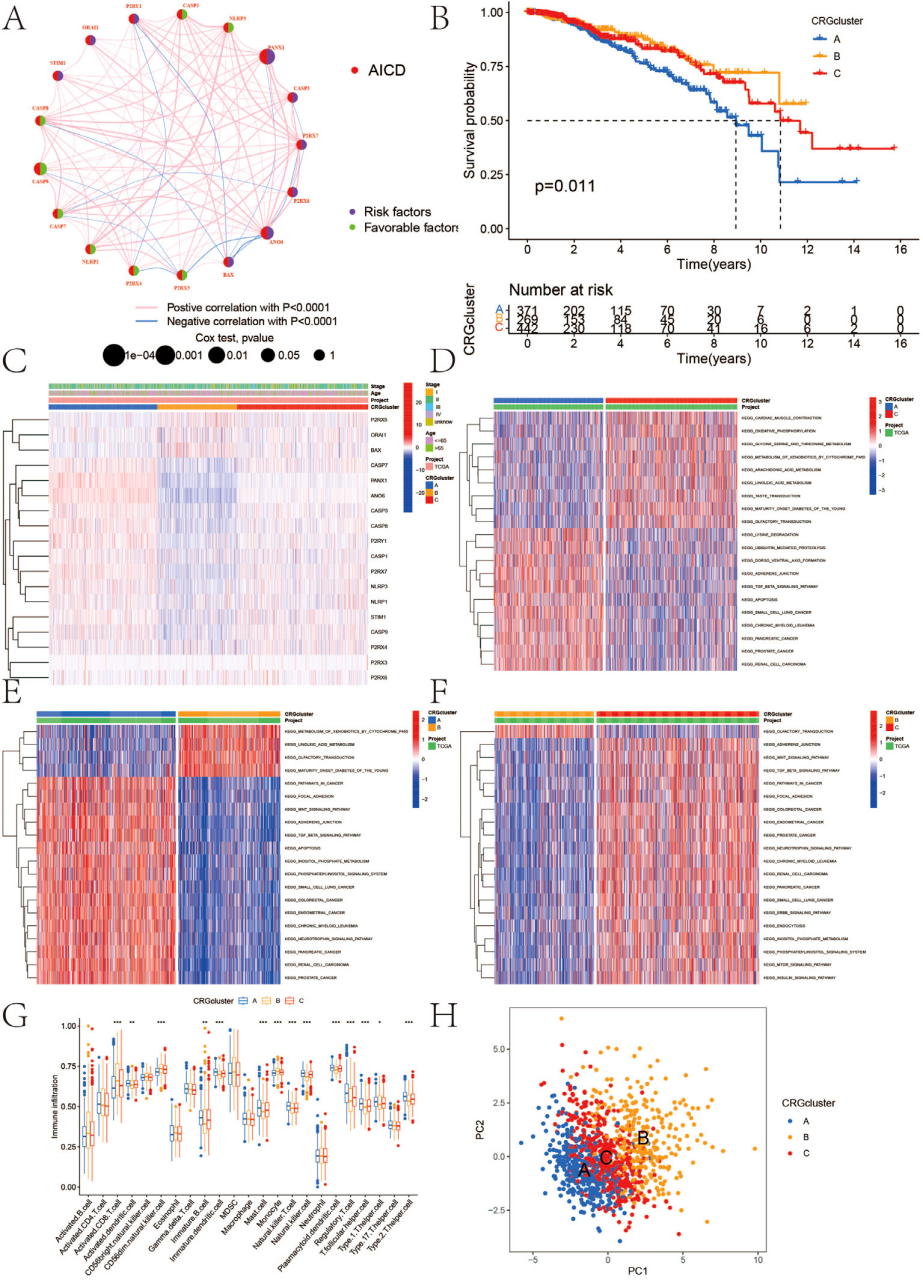
**(A)** Interactions among AICD in BC that are depicted, with red and blue strings indicating positive and negative correlations, respectively; the reflection of the intensity of the correlation in the color shading; **(B)** Kaplan-Meier overall survival curve illustrating AICD subtype specificity; **(C)** Variations in expression levels and clinicopathological features of AICD across different subtypes; **(D–F)** GSVA analysis of pathways among distinct subtypes; **(G)** Correlation of immune cell infiltration levels across various subtypes (ssGSEA); **(H)** Significant transcriptomic differences among subtypes are observed through PCA analysis. (*P < 0.05, **P < 0.01, ***P < 0.001.).

The survival analysis revealed statistically significant differences in the expression of ANO6, CASP9, BAX, P2RX4, CASP1, P2RX3, CASP7, P2RX5, CASP8, P2RX7, PANX1, and STIM1 genes, all of which are associated with prognosis (**Figure 1E**). The findings indicate that high-risk genes such as CASP9, P2RX4, CASP1, P2RX7, P2RX5, and CASP8 correlate with a poorer prognosis. In contrast, ANO6, BAX, P2RX3, CASP7, PANX1, and STIM1 were identified as low-risk genes, correlating with a more favorable prognosis. All 18 AICD-related genes demonstrated a positive relationship, suggesting potential interactions or regulatory roles within the same biological process or pathway. Furthermore, a negative correlation was observed between P2RY1, CASP8, PANX1, CASP9, CASP7, P2RX4, P2RX5, BAX, ANO6, P2RX7, and CASP3, implying the existence of antagonistic or inhibitory relationships within the regulatory processes (**Figure 2A**).

### 3.3 Classification of AICD

**Figure 3:**
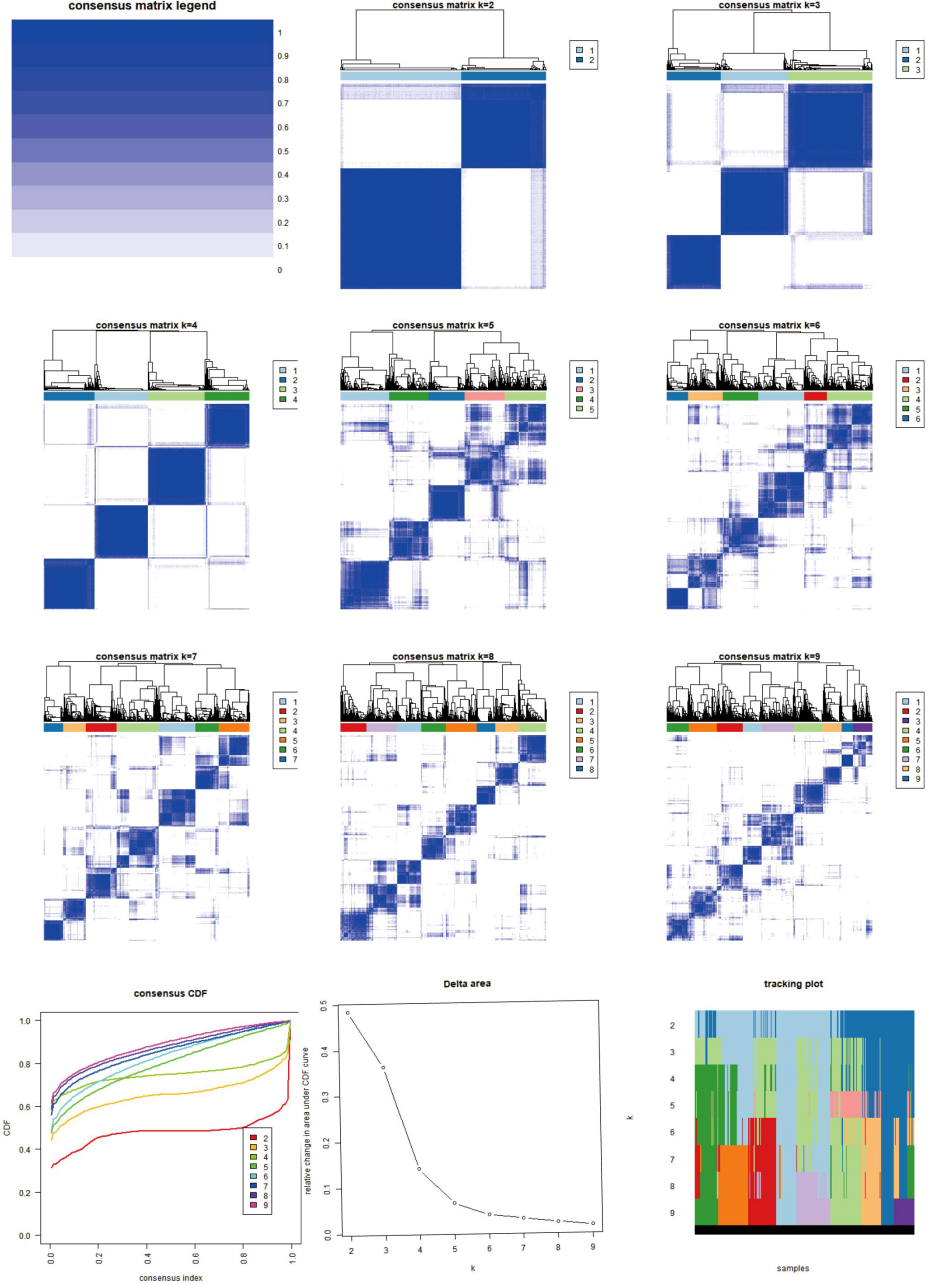
Heat map of two clusters of consensus matrix of AICD. This image illustrates the consensus matrices and their respective heatmaps for varying numbers of clusters (k=2 to k=9), along with the Consensus Cumulative Distribution Function (CDF), Delta area plot, and cluster tracking plot. The consensus matrix heatmaps depict the stability and consistency of data clustering across different k values, with colors transitioning from white to blue indicating consensus levels from low to high. The CDF plot in the lower-left corner demonstrates the cumulative distribution functions for each k value, while the Delta area plot below it quantifies the variations in consensus CDFs across different k values. The cluster tracking plot in the lower-right corner shows how sample cluster assignments evolve as k changes. These visualizations collectively provide a comprehensive perspective for observing and evaluating the stability and adaptability of data clustering under varying numbers of clusters.

The classification results of AICD are illustrated in **Figure 3**. By analyzing 18 genes associated with AICD, we obtained comprehensive sample data, allowing us to categorize all cases into three distinct groups (Group A, Group B, and Group C). The survival analysis, based on this classification, revealed differences in prognosis among the three groups, with Group B exhibiting the most favorable outcomes. Notably, the majority of the 18 AICD-related genes were underexpressed in Group B (**Figures 2B, C**). Furthermore, **Figure 2H** demonstrates that the expression levels of these genes effectively differentiate between samples from Group A, Group B, and Group C. The GSVA analysis results indicated that Groups A, B, and C each displayed distinctively enriched signaling pathways (**Figures 2D-F**). The difference analysis of immune cells showed that Activated.CD8. T.celll; Activated.dendritic.cell; CD56 dim.natural.killer. cell; Immature. B.cell; Immature. dendritic.cell; Mast.cell; Monocyte; Natural.killer. T.cell; Natural.killer.cell, etc. The difference between ABC groups was statistically significant. Among them, 9 immune cell types in group A were highly expressed, 5 immune cell types in group B were highly expressed, and no immune cell types in group C were highly expressed (**Figure 2G**).

### 3.4 Differential analysis of AICD types and enrichment analysis

**Figure 4:**
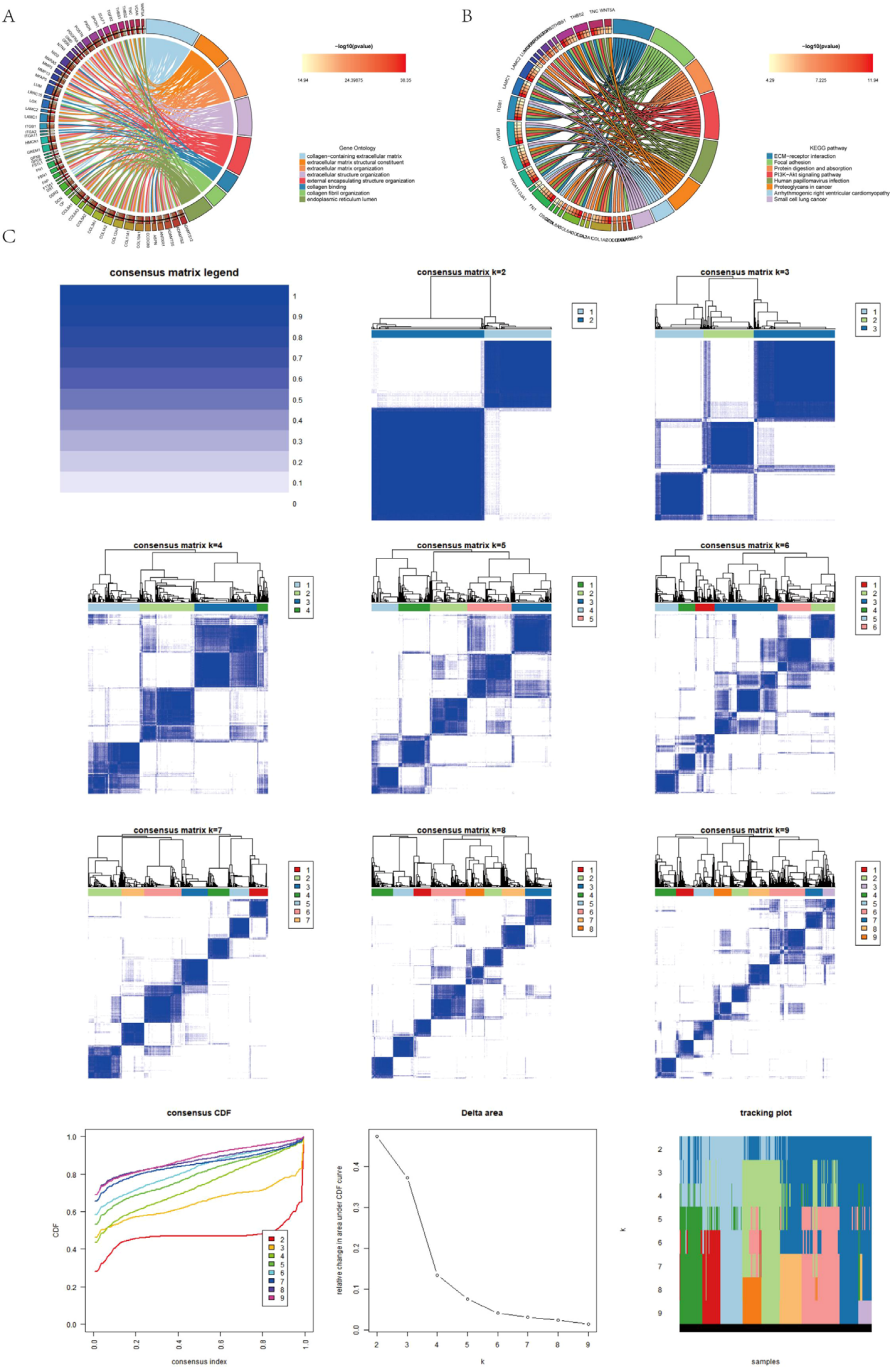
**(A, B)** Conduction of enrichment analysis of differentially expressed genes (DEGs) within two AICD-related subgroups utilizing GO and KEGG methodologies. **(C)** Presentation of a heatmap representing the consensus matrix for differential gene clustering.

Differentially expressed genes were identified in the AICD classification. GO enrichment analysis showed that collagen−containing extracellular matrix, extracellular matrix structural constituent, extracellular matrix organization, etc. (**Figure 4A**). In addition, KEGG pathway analysis showed that ECM−receptor interaction, PI3K−Akt signaling pathway, protein digestion and absorption, etc. (**Figure 4B**).

### 3.5 Differential genotyping

**Figure 5:**
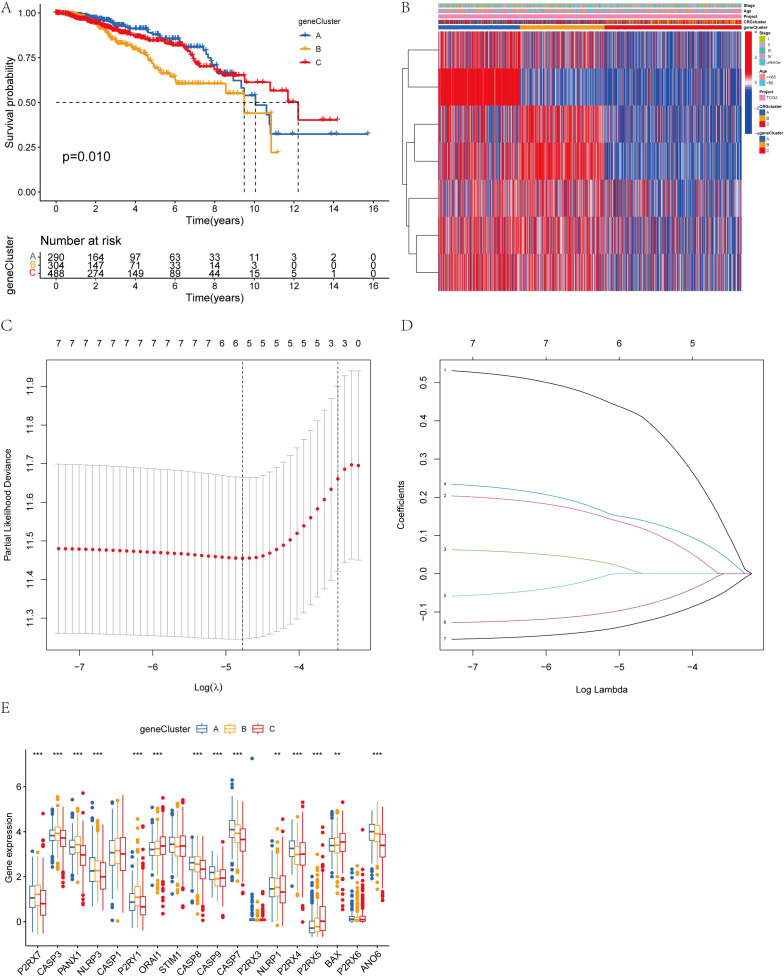
**(A)** Kaplan-Meier overall survival curves for the three gene subtypes; **(B)** Variations in clinicopathological features among the three gene subtypes; **(C, D)** Cross-validation of CRG regression and LASSO regression parameter selection was conducted using LASSO; **(E)** Expression changes of 18 AICD genes across the three gene subtypes. (**P < 0.01, ***P < 0.001.).

To establish more precise prognostic significance, the differential genes in 3.4 were employed for genotyping. Comprehensive sample data enabled the classification of all cases into three distinct subtypes: A, B, and C (**Figure 4C**). Survival analysis based on these subtypes revealed a statistically significant difference in patients’ prognosis among the groups, with Group A demonstrating the most favorable outcome (**Figure 5A**). Notably, within the context of AICD-related genes, the expression levels of most genes in Group A were lower compared to those in Groups B and C (**Figure 5B**). **Figure 5E** further illustrates that the expression levels of these genes can effectively differentiate samples belonging to Groups A, B, and C.

### 3.6 Prognostic model construction

We conducted univariate Cox regression analysis and identified the six genes significantly associated with BC (P<0.05). Building on the critical genes identified through the univariate Cox analysis, we proceeded with LASSO regression and multivariate Cox regression analyses to further refine and identify four key prognostic genes (**Figures 5C, D**). Based on these findings, we developed a prognostic index for all BC patients, and calculated using the following formula:


$$\begin{array}{l} \text{Prognostic }Index=ANO6\times 0.643751453832269+CEMIP\times \\ 0.200172499835835-SLC1A1\times 0.133581567013211-CLIC6\\ \times 0.173118868049522 \end{array}$$

**Figure 6:**
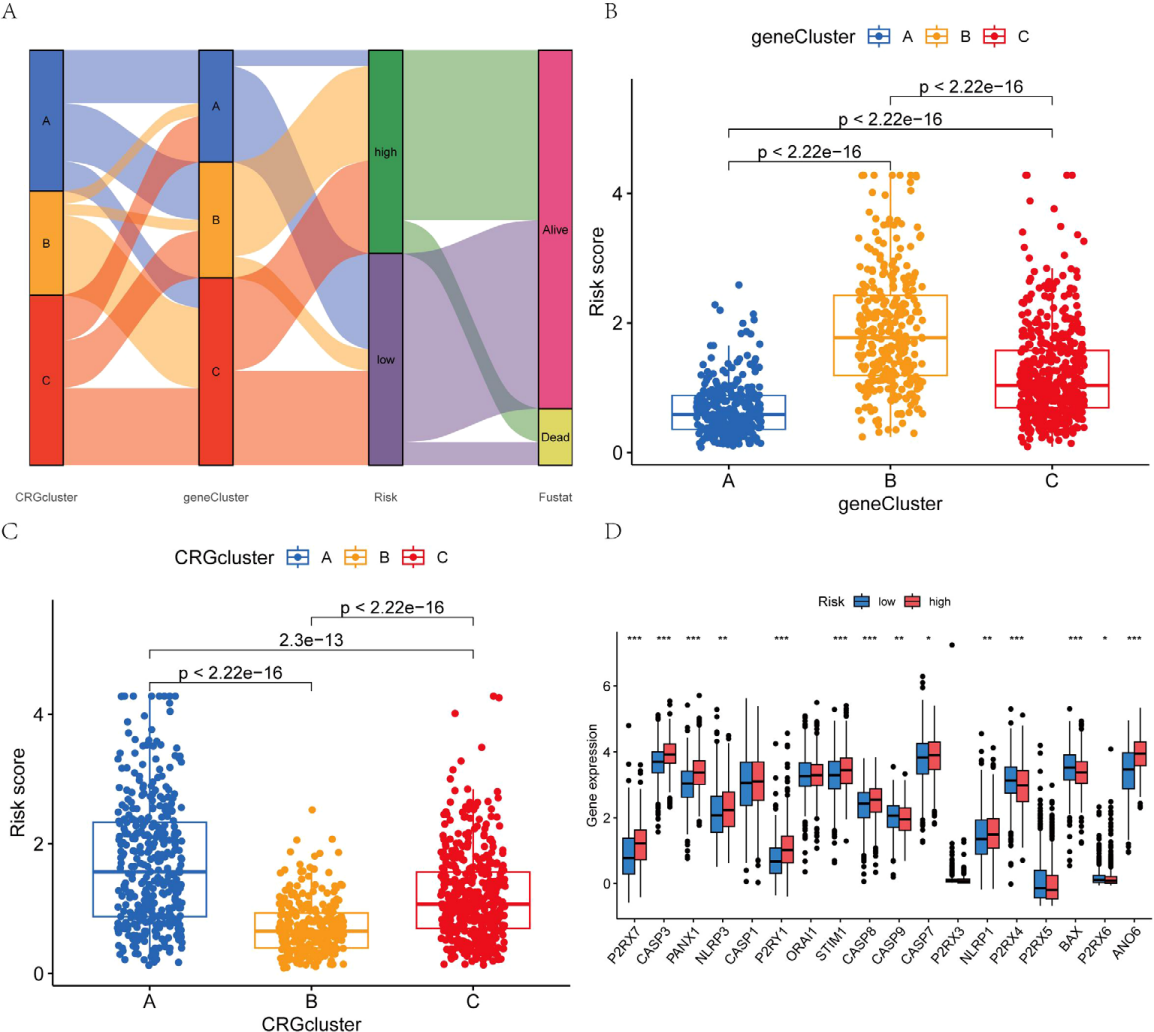
**(A)** Intergroup distribution of subtypes, AICD scores, and survival outcomes; **(B, C)** Variations in AICD scores among BC subtypes; **(D)** Differences in the 18 AICD scores. (*p < 0.05, **p < 0.01, ***p < 0.001.).

Initially, the differential genes were categorized into two distinct sample types based on the expression levels of the mutated genes. Through differential analysis, these genes were further classified into three distinct types. Subsequently, the patients were stratified into two distinct risk groups according to the expression profiles of the differential genes, and a prognostic model was employed to predict individual survival outcomes (**Figures 6A, B**). Among BC subtypes A and B, patients with subtype A exhibited a lower risk. Notable distinctions were observed between patients with genotypes A and B, as well as genotype C, with patients harboring genotype B showing a reduced risk (**Figure 6C**). The genes P2RX7, CASP3, PANX1, NLRP3, CASP1, P2RY1, STIM1, CASP8, CASP7, NLRP1, and ANO6 were highly expressed in the high-risk group, while ORAI1, CASP9, P2RX4, P2RX5, BAX, and P2RX6 showed elevated expression in the low-risk group (**Figure 6D**).

### 3.7 Survival analysis as well as ROC curve

**Figure 7:**
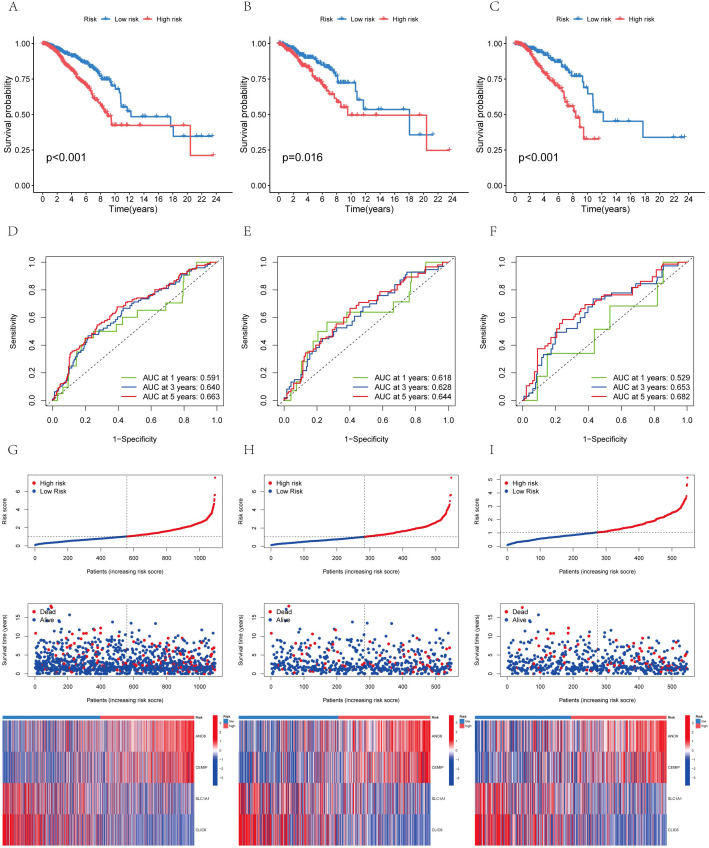
The distribution of patient survival status and AICD scores exhibited variation between the training and test sets **(A)** Aggregate data set; **(B)** Training set; **(C)** Test sets. Receiver operating characteristic (ROC) curve: **(D)** Total data set; **(E)** Training set; **(F)** Test sets. Risk curve: **(G)** Total data set; **(H)** Training set; **(I)** Test sets.

**Figure 8:**
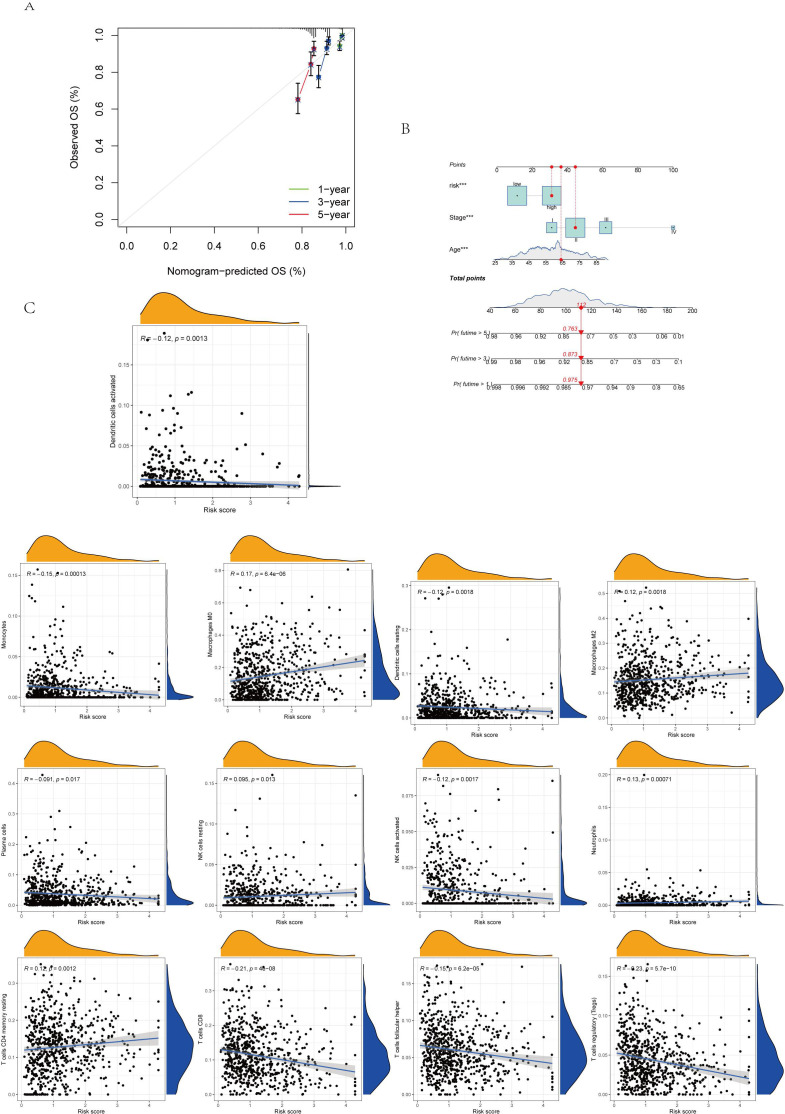
**(A.B)** Column diagram: Find the calibrated curve of the total survival histogram model in the group, and the dashed diagonal line indicates the ideal histogram, while the blue, red, and orange lines indicate the observed 1y, 2y, and 3y histograms. **(C)** Association between immune cell type and AICD score. ***p < 0.001.

Subsequently, utilizing R’s caret package, the patients were randomly allocated to the training group and the test group in a 1:1 ratio. The prognostic differences among the overall risk group, test group and training group were statistically significant (P<0.01), with the prognosis of high-risk groups being generally worse than that of low-risk groups (**Figures 7A-C**). This indicates that the model effectively distinguishes between high-risk and low-risk populations. The area under the curve (AUC) for the training group was higher than that of the test group, with all groups exhibiting an AUC greater than 0.52 (**Figures 7D-F**). BC samples were stratified into two groups based on the median diagnostic risk level. **Figures 7G-I** illustrate the distribution of risk levels, survival status, and survival time of BC patients within these two groups. Additionally, the relative expression levels of four BC-associated genes were presented for each patient, where ANO6 and CEMIP were identified as high-risk genes, and SLC1A1 and CLIC6 as low-risk genes. We evaluated the model’s prognostic accuracy using a standardized test set and a risk score concentration formula for each patient. **Figures 8A, B** display the model’s nomogram, which demonstrates the model’s precision in prognostication.

### 3.8 Immune cell infiltration and Analysis of tumor microenvironment

**Figure 9:**
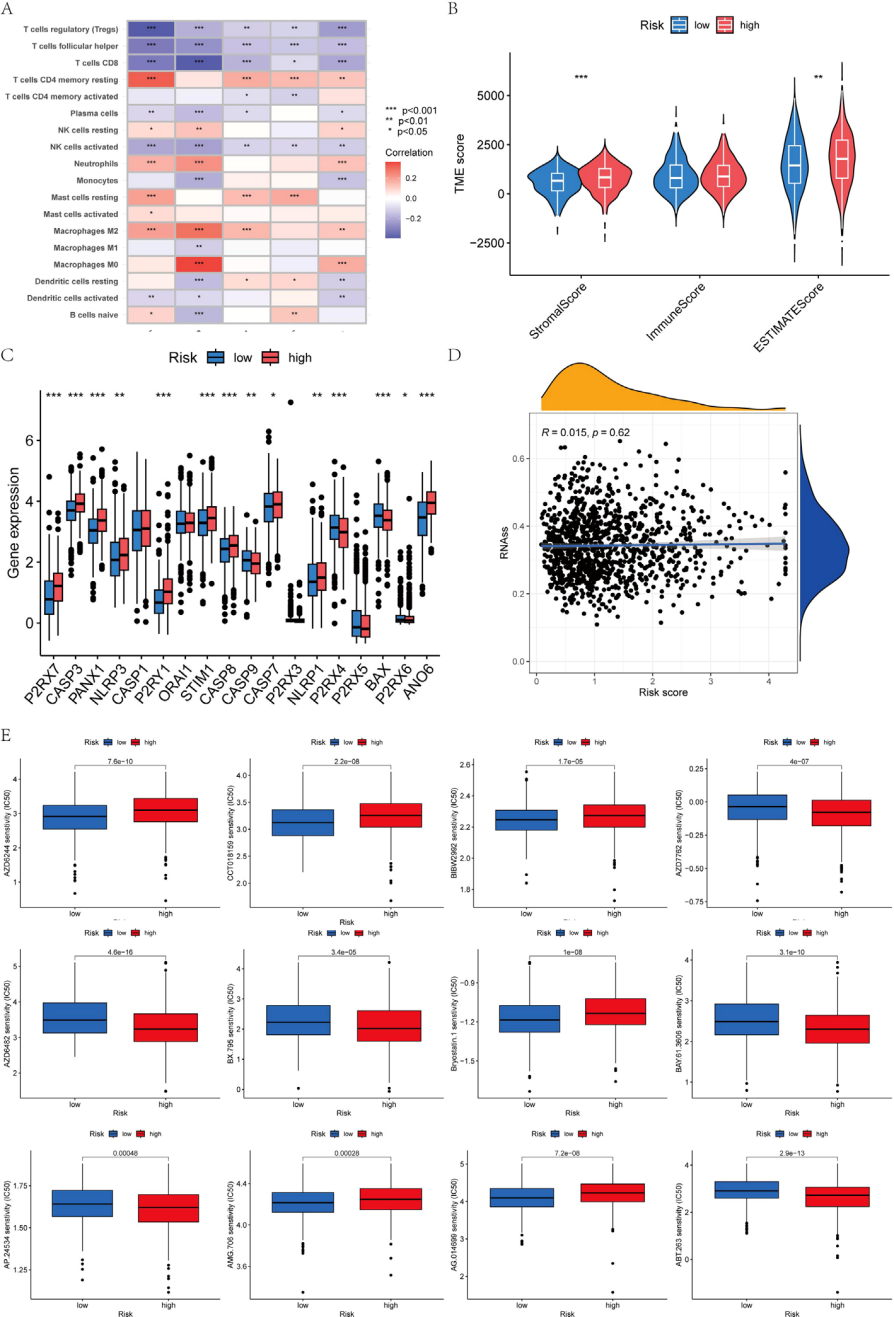
**(A)** Five genes within the model demonstrated an association with the quantity of immune cells. **(B)** Differential analysis of 18 genes; **(C)** Immune score gene mutation analysis; **(D)** Correlation between the cancer stem cell (CSC) index and AICD score; **(E)** Drug sensitivity analysis.

The immune cell correlation analysis revealed that Macrophages M0, M2, and resting CD4 memory T cells were positively correlated with the risk score, indicating an association with higher risk. Conversely, activated and resting dendritic cells, monocytes, neutrophils, activated and resting NK cells, plasma cells, CD8 T cells, and regulatory T cells (Tregs) were negatively correlated with patients’ risk scores (**Figures 8C**, **9A**). In terms of the tumor microenvironment score, the ImmuneScore did not show significant differences between the two distinct populations. However, the StromalScore and ESTIMATE scores demonstrated significant variations between these populations (**Figure 9B**). **Figure 9C** illustrates the mutations in 18 AICD-related genes. Additionally, our stem cell correlation analysis indicated a positive association between stem cells and the risk index, although this correlation was not statistically significant (**Figure 9D**).

### 3.9 Drug sensitivity analysis

**Figure 10:**
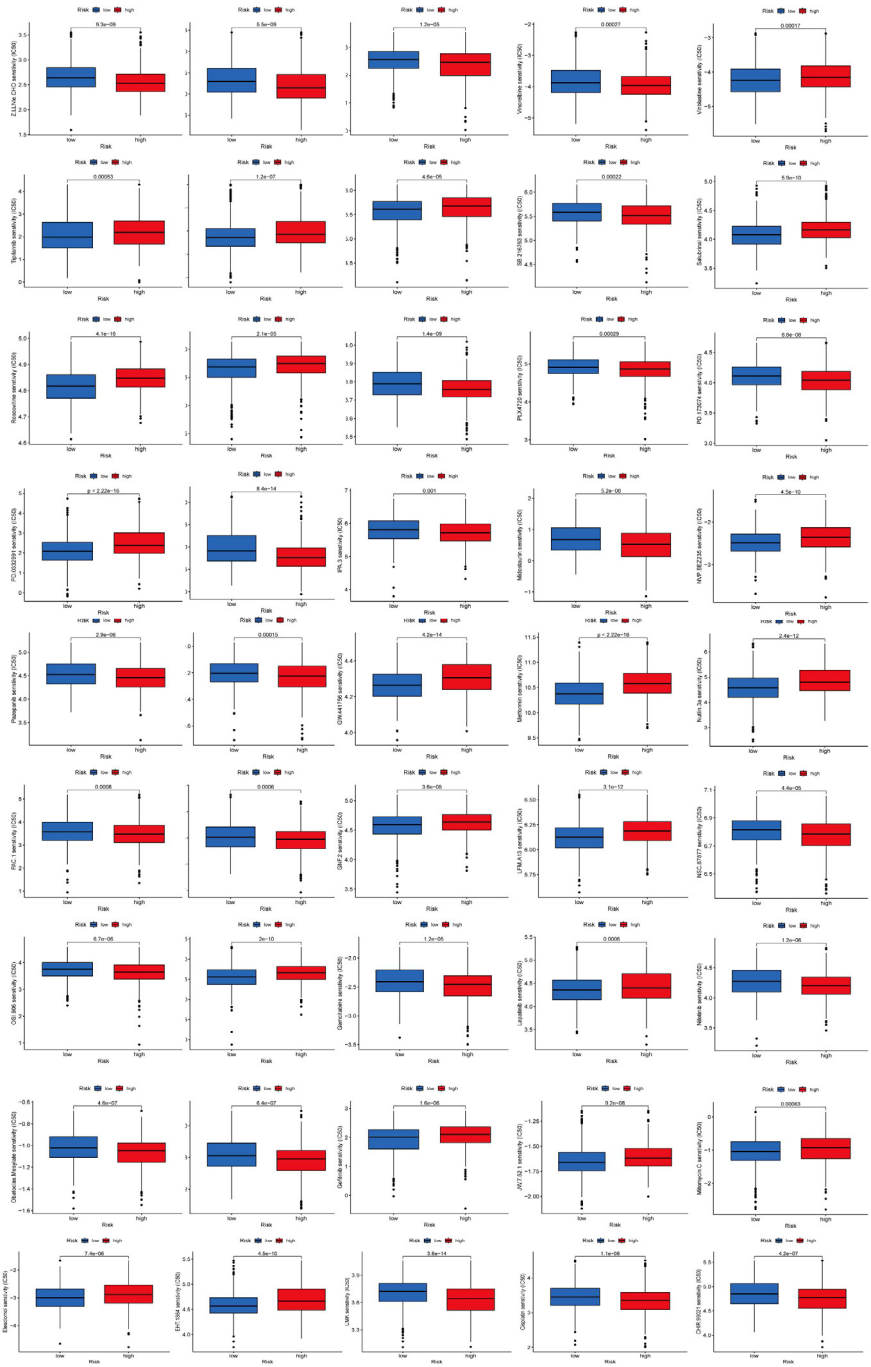
Drug sensitivity analysis.

To identify novel compounds for the treatment of BC based on genetic models, we employed the PRISM algorithm to evaluate the therapeutic response of each sample by assessing the half-maximal inhibitory concentration (IC50) using data from the Cancer Drug Sensitivity Genomics (GDSC) dataset. A total of 57 potential drugs were identified, with significant differences in drug sensitivity observed between the low-risk and high-risk groups. Among these 57 chemotherapeutic agents, 30 exhibited lower IC50 values in the low-risk group compared to the high-risk group (Navitoclax, Ponatinib, Antiplatelet agent, Chk inhibitor, Syk inhibitor, PDK1 inhibitor, Lestaurtinib, GSK-3 inhibitors, An anticancer drug containing platinum, Caspase-1 Inhibitor II, Cell permeability XIAP inhibitor, Macrolides, Inhibitors of topoisomerase II, Gemcitabine, P21 activates kinases, Midostaurin, Nilotinib, Shp2 and Shp1 protein tyrosine phosphatase inhibitors, Obatoclax. Mesylate, IGF-1R inhibitors, Procaspase-3 activator, Pazopanib, FGFR1 inhibitors, B-RafV600E inhibitor, ARFGAP1 inhibitors, GSK-3 inhibitors, Vinorelbine, Shikonin, Selective potent inhibitors of ERK5 and LRRK, Gamma-secretase inhibitors). Conversely, 27 drugs (Rucaparib, Motesanib Diphosphate, Selumetinib, Afatinib, PKC stimulant, 3, 4-diarylpyrazole resorcinol, Rac family GTPase inhibitors, Oxidative stress inducer, Erlotinib, EGFR-TK inhibitors, Bcr-Abl inhibitors, TrkA inhibitor, MELK inhibitor, Lapatinibs, BTK, JAK2, PLK inhibitors, Metformin, Mitomycin.C, p53-MDM2 inhibitors, Dactolisib, Palbociclib, ATP competitive selective inhibitors of CDK1, Roscovitine, Salubrinal, Selective p90 ribosome S6 kinase (RSK) inhibitors, Temsirolimus, Tipifarnib, Vinblastine) showed higher IC50 values in the high-risk group, indicating that patients in the low-risk group may be more responsive to chemotherapy, which could contribute to a better prognosis (**Figures 9E**, **10**).

### 3.10 Cell activity

**Figure 11:**
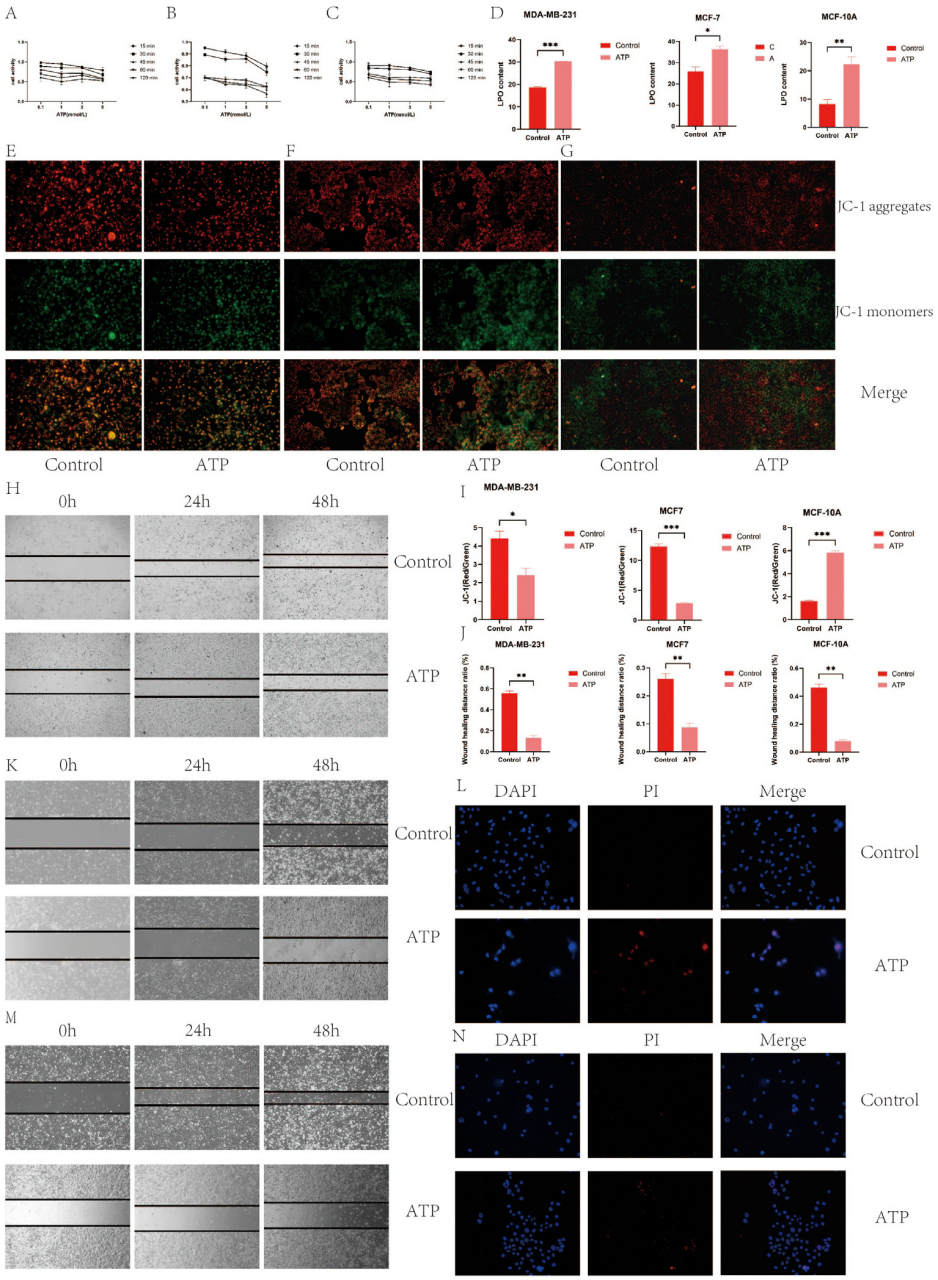
**(A)** MDA-MB-231 cell activity; **(B)** MCF7 cell activity; **(C)** MCF-10A cell activity; **(D)** ATP increased the level of intracellular LPO; **(E)** ATP decreased mitochondrial membrane potential of MDA-MB-231 BC cells; **(F)** ATP decreased mitochondrial membrane potential of MCF7 BC cells; **(G)** ATP decreased mitochondrial membrane potential of MCF-10A BC cells; **(I)** Quantization of mitochondrial membrane potential histogram; **(H)** MDA-MB-231 cell migration; **(J)** Histogram of quantified results of scratch experiments; **(K)** MCF7 cell migration **(M)** MCF-10A cell migration; **(G)** Quantified histograms of cell migration; **(L)** MDA-MB-231 immunofluorescence detection of apoptosis; **(N)** MCF7 immunofluorescence detection of apoptosis. (*p < 0.05, **p < 0.01, ***p < 0.001.).

**Figures 11A-C** illustrate the variations in cell viability across different ATP concentrations (0.1 mmol/L, 1 mmol/L, 3 mmol/L, 5 mmol/L) within five experimental groups, each subjected to different treatment durations (15 minutes, 30 minutes, 45 minutes, 60 minutes, 120 minutes). As ATP concentration increased, a general decline in cell viability was observed at each time point. The 15-minute treatment group consistently exhibited higher cell viability across all ATP concentrations, indicating superior cell activity. Conversely, the 120-minute treatment group consistently demonstrated the lowest cell viability across all ATP concentrations, reflecting the most diminished cell activity. Based on a screening criterion of 50-60% cell viability, the MDA-MB-231 cell line was selected at 0.1 mmol/L for 120 minutes, MCF7 was selected at 1 mmol/L for 45 minutes, and MCF-10A was selected at 1 mmol/L for 120 minutes.

### 3.11 Elevation of intracellular LPO levels by ATP

Compared to the control group, both the ATP-treated group and the LPO-treated group exhibited a significant increase in content levels (P<0.05), as depicted in **Figure 11D**.

### 3.12 Reduction of the mitochondrial membrane potential in BC cells by ATP

The immunofluorescence staining results revealed that in MDA-MB231 and MCF7 cells, the ATP group exhibited the most intense green fluorescence and the weakest red fluorescence, indicating a decreasing trend in mitochondrial membrane potential during the early stages of apoptosis. Quantitative analysis demonstrated that the mitochondrial membrane potential in the control group was significantly higher than in the ATP group (P<0.01), as shown in **Figures 11E–G, I**. This suggests that ATP exerts a detrimental effect on the mitochondrial membrane potential in MDA-MB231 and MCF7 BC cells. Conversely, in MCF-10A cells, the mitochondrial membrane potential in the control group was lower than that in the ATP group, indicating that ATP has a protective effect on the mitochondrial membrane potential of MCF-10A cells in BC.

### 3.13 Inhibition of the migration of BC cells by ATP

The scratch assay results indicated that the scratch healing rate in the ATP group was significantly lower than that in the control group (P < 0.01), as illustrated in **Figures 11H, J, K, M**. These findings suggest that ATP effectively impedes the migration of BC cells.

### 3.14 s Promotion of apoptosis of BC cells by ATP

**Figure 12:**
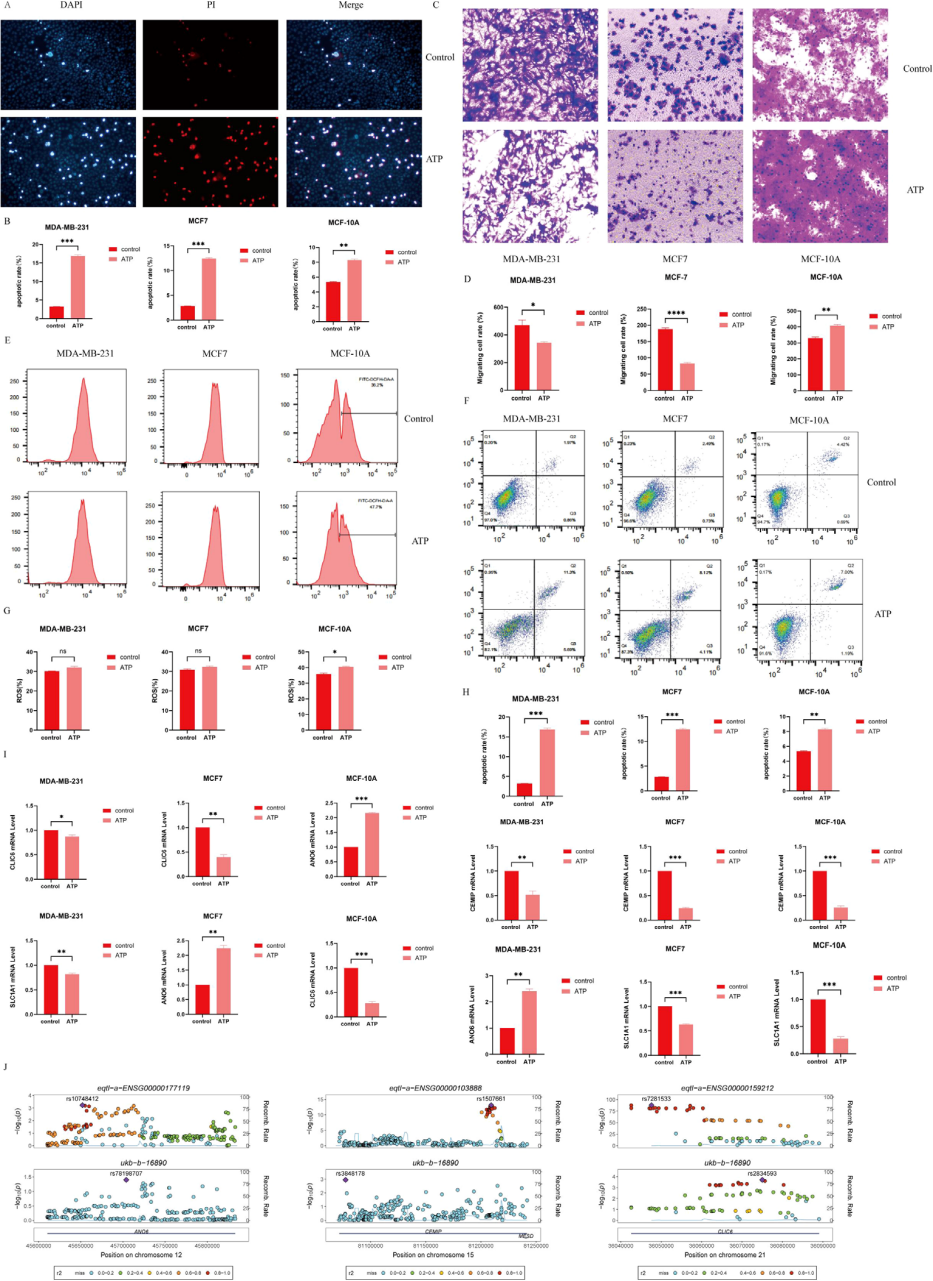
**(A)** Detection of Apoptosis by immunofluorescence; **(B)** Cell apoptosis quantifying histogram by immunofluorescence detection; **(C)** Cell migration; **(D)** Histogram of quantified cell migration; **(E)** Use of flow cytometry to detect ROS content; **(G)** Quantification of cell ROS content by flow cytometry histogram;**(F)** Detection of the apoptosis rate was detected by flow cytometry; **(H)** Measurement of cell apoptosis rate by flow cytometry; **(I)** Detection of the expression levels of ANO6, CEMIP, CLIC6 and SLC1A1 by RT-qPCR; **(J)** Genetic variation -eQTL-GWAS colocalization. (*P < 0.05, **P < 0.01, ***P < 0.001, ****P < 0.0001).

TUNEL staining results (**Figures 11L, N**, **12A, B**) demonstrated that ATP significantly enhances apoptosis compared to the untreated group (P<0.01).

### 3.15 Inhibition of the migration of BC cells by ATP

In MCF7 and MDA-MB231 cells, the number of migrating cells in the ATP group was significantly reduced compared to the Control group (P < 0.05). Conversely, in MCF-10A cells, the number of migrating cells in the ATP group was markedly higher than that in the Control group (P < 0.01), indicating statistical significance. Refer to **Figures 12C, D** for details.

### 3.16 Promotion of apoptosis of BC cells by ATP

Flow cytometry (**Figures 12F, H**) revealed that the apoptosis rate in the ATP group was significantly elevated compared to the control group (P<0.01).

### 3.17 Promotion of ROS production in MCF-10A cells by ATP

In comparison to the control group, the reactive oxygen species (ROS) content in MCF-10A cells was significantly elevated following ATP intervention (P<0.05) (**Figures 12E, G**). Although the ROS levels in MCF7 and MDA-MB231 cells also exhibited an increase, this change was not statistically significant.

### 3.18 Detection of the expression of ANO6, CEMIP, CLIC6 and SLC1A1 in BC cells by RT-qPCR

In comparison to the control group, the expression levels of CLIC6, SLC1A1, and CEMIP were significantly reduced in the ATP intervention group, while the expression level of ANO6 was notably elevated (**Figure 12I**).

### 3.19 Genetic variation -eQTL-GWAS colocalization

ANO6, CEMIP, and CLIC6 exhibit shared genetic causal variation with BC, whereas SLC1A1 does not demonstrate such a genetic association with the disease (**Figure 12J**).

## 4 Discussion

BC has emerged as a significant global public health issue, representing the most prevalent cancer among women. In 2020, it was estimated that there were 2.3 million new cases and over 685,000 fatalities worldwide. Despite substantial improvements in survival rates over the past two decades, the incidence of this malignancy continues to escalate on a global scale (1). Current treatment modalities are frequently accompanied by adverse side effects, recurrence, and a diminished quality of life. While traditional therapies, such as surgery and chemotherapy, have been utilized for many years, their detrimental impacts on normal cells and tissues remain a critical limitation, ultimately resulting in the death of cancer cells, potentially through mechanisms such as apoptosis, necrosis, or autophagy (6). For instance, Vinettoc (VCX), a selective BCL-2 inhibitor, influences the molecular mechanisms governing the growth and proliferation of MDA-MB-231 cells in triple-negative BC (TNBC) by inducing apoptosis, causing cell cycle arrest, and promoting autophagy (7).

There is an increasing body of evidence indicating that ferroptosis and cuproptosis — distinct forms of regulated cell death — are highly effective in inhibiting tumor growth. Targeting ferroptosis presents a promising anticancer strategy. The recent identification of ferroptosis inducers, coupled with a comprehensive elucidation of their regulatory mechanisms and associated genes, has established a robust foundation for the development of cancer treatment strategies that exploit ferroptosis (8, 9). Notably, the extracellular ATP (eATP) released in response to copper stress serves as a diffusion signal that can effectively mitigate cell death induced by such stress. When copper stress in chamber A triggers the death of plant cells in chamber B, the transfer of ATP from chamber A likely plays a pivotal role in counteracting this cell death. Consequently, studies have demonstrated that eATP released under conditions of copper stress can function as a diffusion signal to alleviate copper-induced cell death (10). Ferroptosis is an increasingly scrutinized pathway of cell death, particularly in the context of overcoming drug resistance in tumor therapies. Extracellular ATP is critical in enhancing iron uptake and activating mitochondrial ATP production in iron-rich environments. The survival benefit conferred by ATP-Fe complexes in iron-sensitive inert cell lines further underscores the intricate relationship between ATP and iron (11). In summary, whether through ferroptosis or cuproptosis, cells release ATP, leading to elevated extracellular ATP levels. As a mediator of cell death release, ATP provides profound insights into the mechanisms underlying cellular demise. This study seeks to delve deeper into the innovative mechanisms and therapeutic potential of AICD, with the objective of enhancing the cure rate for patients. To our knowledge, there has been scant research investigating the potential relationship between AICD and BC. Our findings indicate that BC tissues exhibit differential expression of genes associated with AICD compared to normal tissues. Moreover, the varying expression levels of these genes among individuals may provide prognostic or predictive insights for BC patients. In the context of BC, the upregulation of P2X7R has been shown to facilitate tumor invasion and metastasis by activating the protein kinase B (AKT) signaling pathway, promoting epithelial-mesenchymal transition (EMT), regulating the generation of extracellular vesicles (EVs), and influencing the expression of the inflammation-related protein cyclooxygenase-2 (COX-2) (12). Additionally, caspases (CASP), encoded by the ASP family of genes, play a crucial role in the induction, transduction, and amplification of apoptotic signals. Notably, CASP9 functions as the initiator caspase in the mitochondrial cell death pathway, and its upregulation can significantly trigger apoptosis in various cell types. The expression level of CASP9 is also closely correlated with the prognosis and immune cell infiltration in BC (13).

A plethora of studies has elucidated the association between ATP and copper in the development, proliferation, and carcinogenesis of human tumor cells. Nonetheless, a more profound exploration of specific mechanisms—including the initiation of tumor stem cells, tumor growth, and metastasis—is imperative to establish a definitive causal relationship between ATP and human malignancies. Currently, the ramifications of AICD and its influence on immune infiltration characteristics remain inadequately understood. Our findings indicate that high-risk genes such as CASP9, P2RX4, CASP1, P2RX7, P2RX5, and CASP8 exhibit a robust correlation with poorer prognoses. Conversely, genes such as ANO6, BAX, P2RX3, CASP7, PANX1, and STIM1 have been identified as low-risk genes associated with a more favorable prognosis. Research has demonstrated that the activation of p38 MAPK and the enhancement of intracellular calcium uptake are intricately linked to the ligand-gated ion channel P2RX4.

P2X receptors represent a class of ATP-gated ion channels comprised of seven known members (P2X1 to P2X7), which play a pivotal role in the rapid responses of various mammalian cells—including white blood cells, vascular smooth muscle cells, sensory neurons, and endothelial cells—to ATP as a signaling molecule. Calcin, in particular, induces an elevation in intracellular calcium levels through a P2RX4-dependent mechanism, thereby mediating apoptosis in breast and cervical cancer cell lines (14). The compound not only facilitates an increase in intracellular calcium concentration but also enhances the mRNA expression of the ATP receptor P2RX4 and the phosphorylation level of p38. Significantly, the application of BAPTA-AM can effectively obstruct the rise in intracellular calcium levels, while the antagonist 5-BDBD can efficiently inhibit the expression of P2RX4. Furthermore, SB203580 has the capacity to block the phosphorylation of p38, collectively eliminating the apoptotic activity induced by calcin (14).

By analyzing 18 genes associated with AICD, we categorized all cases into three distinct groups (Groups A, B, and C). Notably, Group B exhibited the most favorable outcomes, characterized by the underexpression of the majority of the 18 genes linked to AICD. Furthermore, differential analysis of immune cell populations revealed variations in the expression of activated CD8+ T cells, activated dendritic cells, CD56^dim natural killer cells, immature B cells, immature dendritic cells, mast cells, monocytes, and natural killer T cells. Specifically, nine immune cell types were found to be highly expressed in Group A, five in Group B, while no significantly elevated immune cell types were detected in Group C. Subsequently, we identified gene subtypes among the three AICD classifications using differentially expressed genes (DEGs). In this context, we also developed a prognostic AICD score and validated its predictive capacity. Compared to low-risk patients, those with high AICD scores demonstrated significant disparities in overall survival, clinical characteristics, mutation profiles, tumor microenvironment (TME) dynamics, cancer stem cell (CSC) indices, and drug resistance. To enhance practicality and facilitate the application of the AICD score, we created a nomogram based on patient characteristics and the AICD score. This prognostic model is anticipated to further elucidate the molecular mechanisms underlying BC and offer innovative insights for therapeutic strategies.

Moreover, through LASSO regression and multivariate Cox regression analysis, we derived the following prognostic indices: Prognostic Index = ANO6 × 0.643751453832269 + CEMIP × 0.200172499835835 - SLC1A1 × 0.133581567013211 - CLIC6 × 0.173118868049522. Notably, the expression levels of ANO6 were significantly diminished in BC (BC) tissues compared to normal breast tissue, and its overexpression was found to independently predict unfavorable overall survival (OS) among BC patients. Limited cubic spline analysis further revealed a linear relationship between ANO6 expression and OS. Interestingly, menopausal status emerged as an interaction factor influencing ANO6’s association with OS. Additionally, ANO6 was implicated in stroma-related pathways, with its upregulation showing a significant correlation with elevated stromal scores and macrophage polarization. Furthermore, ANO6 expression levels were closely associated with the expression of immune checkpoints, tumor mutation load, and microsatellite instability scores (15).In contrast, CEMIP expression was markedly elevated in BC tissues compared to normal tissues, with higher mRNA levels of CEMIP correlating with poorer survival outcomes. CEMIP and its co-expressed genes may be involved in biological processes associated with BC progression, such as hyaluronic acid biosynthesis and catabolism (16). The expression levels of SLC1A1 and SLC1A3 in non-invasive MCF-7 BC cells were significantly lower than those observed in the highly invasive MDA-MB-231 BC cells (17). Additionally, a study constructed a prognostic profile consisting of nine genes associated with ferroptosis, including ALOX15, CISD1, CS, GCLC, GPX4, SLC7A11, EMC2, G6PD, and ACSF2 (18). BC represents the subtype with the most dismal prognosis in BC, and the utilization of 15 ferroptosis-related genes may influence TNBC prognosis by modulating the tumor microenvironment (19). Based on multivariate Cox regression analysis, six Cuproptosis-related genes (PGK1, RPL14, PRDX1, PSME1, MAL2, and SURF4) were identified using Akaike Information Criterion (AIC) values from 22 OS-related genes to establish a prognostic model (20).

The correlation analysis of immunocells revealed that M0 and M2 macrophages, as well as resting CD4 memory T cells, exhibited a significant positive correlation with risk scores, indicating that these cell types are associated with elevated risk levels. Conversely, active and resting dendritic cells, monocytes, neutrophils, active and resting natural killer (NK) cells, plasma cells, CD8+ T cells, and regulatory T cells (Tregs) demonstrated a negative association with risk scores. Given the successful implementation of immunotherapy in BC treatment, a thorough investigation of the tumor microenvironment and immune cell infiltration will yield crucial insights into the innovative pathways and mechanisms underlying BC immunotherapy.

Extracellular ATP enhances chemoresistance in BC through the HIF-1α signaling pathway (21). In a culture system utilizing human BC T47D cells, the addition of ATP (at concentrations exceeding 0.1 mM) resulted in a pronounced inhibition of cellular proliferation. Subsequent analyses revealed that this inhibitory effect was specific to ATP and closely correlated with its concentration. Notably, the growth suppression persisted for at least three days, despite the complete metabolism of ATP and its hydrolysis products within 24 hours. Furthermore, conditioned media derived from ATP-treated cultures (CM+) exhibited the capacity to inhibit the proliferation of cells that had not been exposed to ATP (22). In the absence of the MCF-10A cell line, taxol-treated BC cell lines demonstrated a dose-dependent decline in cell viability, accompanied by a significant increase in extracellular ATP (eATP) levels. These effects were reversed by specific antagonists of P2 receptors, resembling the observations made with eATP enzyme inhibitors. All assessed cell lines expressed P2RX4 and P2RX7 at mRNA, protein, and cell surface levels. These findings underscore the pivotal role of eATP in modulating the chemotherapeutic response of TNBC cell lines, highlighting its potential to enhance the efficacy of chemotherapy regimens against TNBC (23).The antitumoral activity of ATP was first established in the early 1980s, when exogenous ATP was found to inhibit the proliferation of pancreatic and colon cancer cells. Since then, numerous studies have elucidated the inhibitory effects of ATP across various cancer types, including prostate, breast, colon, liver, ovarian, colorectal, esophageal cancers, melanoma, and leukemia. Clinical trials have demonstrated that intravenous ATP administration can improve survival rates in patients with advanced cancers. While many studies suggest that ATP exerts its anticancer effects through binding to purinergic receptors, some reports indicate that ATP and its mediated purinergic signaling may promote tumorigenesis in prostate and BC cells. This contradiction was clarified in our recent publication, which demonstrated that ATP exerts a biphasic effect on BC cells (24). Specifically, ATP inhibits the migration and bone metastasis of BC by downregulating CXCR4 and the purinergic receptor P2Y11 (25). However, extracellular ATP also promotes BC invasion and chemoresistance via the SOX9 signaling pathway (26).

Research on the effects of P2X7 agonists has illuminated the critical role of purinergic receptors for extracellular ATP in suppressing the growth and migration of cancer cells. Intriguingly, elevated levels of ATP were found to actually enhance the proliferation and migration of BC cells. In contrast, no such deleterious effects were observed with high concentrations of ATPγS, a non-hydrolyzable ATP analogue, which demonstrated a dose-dependent inhibitory effect on BC cell viability. Furthermore, when compared to ATP, adenosine significantly augmented the migratory capacity of BC cells, an effect that could be effectively blocked by adenosine receptor antagonists. Collectively, these findings elucidate the biphasic nature of ATP’s impact on BC cells.

It is noteworthy that pertinent literature has demonstrated that extracellular ATP can activate hypoxia-inducing factor (HIF) signaling pathways, leading to the upregulation of hypoxia-inducing factor 1/2α (HIF-1/2α). Research has revealed that the molecules implicated in the ATP-HIF-2α signaling cascade are significantly overexpressed in human BC tissues and correlate with a poor prognosis (27). Furthermore, extracellular ATP is implicated in the promotion of chemotherapy resistance in BC via HIF-1α signaling, with concentrations of 100 μM extracellular ATP exhibiting the most pronounced anti-apoptotic effects during chemotherapeutic interventions (21). These findings suggest that ATP exerts a detrimental impact on BC, facilitating both the emergence and progression of the disease concurrently with drug treatment, which stands in contrast to the outcomes observed with ATP administered in isolation in this study.

Extracellular ATP is synthesized by S100A4 in cancer cells and fibroblasts, facilitating the migration and metastasis of BC cells, although the specific concentrations are not elucidated in the immunological context of relevant studies (28). Moreover, the ATP-mediated inhibition of cell proliferation is dose-dependent, requiring an optimal concentration of [Ca^2+^] to elicit this effect. Notably, treatment with ATP concentrations resulted in pronounced apoptotic characteristics in two distinct BC cell lines (29). One investigation demonstrated that extracellular ATP activates a plethora of signaling pathways and enhances c-fos gene expression in MCF-7 BC cells in response to growth factors. Furthermore, extracellular nucleotides synergistically interact with growth factors to augment the expression of genes implicated in the proliferation of MCF-7 cells through the activation of specific purinergic receptors, thereby underscoring their potential as critical targets for the prevention of tumor progression in BC. This study also indicated that extracellular ATP promotes EGF-induced transcription of c-fos (30). These findings are in concordance with those of Ben Spungin, which reveal that exogenous ATP induces growth inhibition in BC cells (22).

This study presents several significant contributions to the field. Firstly, it stands as a pioneering investigation that explicitly delineates a subtype correlated with AICD and formulates a predictive model based on this mechanism in BC. Given that the pathway of AICD markedly diverges from other established cell death mechanisms, it opens up entirely novel avenues for therapeutic strategies in oncology. Secondly, this research employs a diverse array of methodologies and databases to bolster the reliability of its findings. We meticulously defined subtypes associated with BC and developed predictive models for screening and validation, thereby enhancing the methodological rigor of the study. Nevertheless, certain limitations must be acknowledged. Data pertaining to several critical clinical factors—such as surgical interventions, chemoradiotherapy, and radiation therapy—are incomplete, and these variables may exert a substantial influence on the prognosis related to immune responses and drug sensitivity.

## 5 Conclusion

In this study, we systematically analyzed the prognostic significance of AICD-related genes in BC, exploring their correlation with the tumor microenvironment and clinical characteristics to develop a robust prognostic prediction model. Additionally, we examined the potential of AICD as a biomarker for therapeutic response. Our findings underscore the clinical relevance of AICD, providing a solid foundation for further investigations into the diagnosis and personalized treatment of BC patients. Moreover, we highlighted the complex role of extracellular ATP, which is typically released from dying or damaged cells to signal cellular stress to adjacent cells. Under normal physiological conditions, this release recruits immune cells to the site of injury for phagocytosis and degradation of necrotic cells. However, at elevated concentrations, ATP is metabolized to AMP and subsequently to adenosine, a process facilitated by exonucleotidases. The resulting extracellular adenosine exerts an immunosuppressive effect, inhibiting the inflammatory response. Notably, exogenous ATP has been shown to play a protective role in BC, further complicating its functional implications in the disease’s pathology.

Looking ahead, future research should focus on developing novel therapeutic agents that target the AICD and ATP-adenosine pathways, aiming to modulate their effects on the immune response and tumor progression. Such investigations could lead to innovative treatment strategies that not only enhance patient outcomes but also redefine the therapeutic landscape of BC. By deepening our understanding of these complex interactions, we can pave the way for more effective, personalized therapies that align with individual patient needs, ultimately improving the management of BC.

## Data Availability

The raw data supporting the conclusions of this article will be made available by the authors, without undue reservation.
